# Basal lamina remodeling at the skeletal muscle stem cell niche mediates stem cell self-renewal

**DOI:** 10.1038/s41467-018-03425-3

**Published:** 2018-03-14

**Authors:** Shantisree Sandeepani Rayagiri, Daniele Ranaldi, Alexander Raven, Nur Izzah Farhana Mohamad Azhar, Olivier Lefebvre, Peter S Zammit, Anne-Gaëlle Borycki

**Affiliations:** 1 Department of Biomedical Science, University of Sheffield, Firth Court, Western Bank, Sheffield S10 2TN UK; 2 Inserm U1109 MN3T, F-67200 Strasbourg, France; 3 Université de Strasbourg, F-67000 Strasbourg, France; 4 LabEx Medalis Université de Strasbourg, F-67000 Strasbourg, France; 5 Fédération de Médecine Translationnelle de Strasbourg (FMTS), F-67000 Strasbourg, France; 6 Randall Centre for Cell and Molecular Biophysics, Faculty of Life Sciences & Medicine King’s College London, New Hunt’s House, Guy’s Campus, London, SE1 1UL UK; 7 Biotherapeutics Development Unit, Cancer Research UK, Clare Hall laboratories, Blanche Lane, South Mimms, Hertfordshire, EN6 3LD UK; 8 MRC Centre for Regenerative Medicine, SCRM Building, University of Edinburgh, 5 Little France Drive, Edinburgh, EH16 4UU UK; 9 Oxford Publishing (Malaysia), Shah Alam, 40150 Selangor Darul Ehsan Malaysia

## Abstract

A central question in stem cell biology is the relationship between stem cells and their niche. Although previous reports have uncovered how signaling molecules released by niche cells support stem cell function, the role of the extra-cellular matrix (ECM) within the niche is unclear. Here, we show that upon activation, skeletal muscle stem cells (satellite cells) induce local remodeling of the ECM and the deposition of laminin-α1 and laminin-α5 into the basal lamina of the satellite cell niche. Genetic ablation of laminin-α1, disruption of integrin-α6 signaling or blocking matrix metalloproteinase activity impairs satellite cell expansion and self-renewal. Collectively, our findings establish that remodeling of the ECM is an integral process of stem cell activity to support propagation and self-renewal, and may explain the effect laminin-α1-containing supports have on embryonic and adult stem cells, as well as the regenerative activity of exogenous laminin-111 therapy.

## Abstract (web summary)

Extracellular matrix (ECM) remodelling is thought to have effects on muscle stem cells that support muscle homeostasis. Here the authors show ECM remodeling controls satellite cell self-renewal through deposition of laminin-α1 into the satellite cell niche.

## Introduction

Substantial progress has been made in understanding the molecular and cellular control mechanisms of embryonic, germline, and adult stem cell activity. The recognition that stem cell activity does not involve solely intrinsic factors, but also depends on extrinsic cues provided by the niche is a major insight into the regulatory events underlying stem cell function and tissue homeostasis^1^. Despite a focus on the niche support cells and the secreted factors they produce, the role of the extra-cellular matrix (ECM) and its signaling function in the stem cell niche is mostly unexplored. Tissue homeostasis in skeletal muscles relies on the activity of muscle-specific stem cells called satellite cells (SCs)^2–5^, which are mono-nucleated cells that express the paired homeodomain transcription factor Pax7 (and in some cases Pax3)^6,7^, and are normally mitotically quiescent. Upon activation caused by exercise, injury or disease, SCs execute a myogenic program, reminiscent of that occurring during embryogenesis, which culminates with the fusion of SC-derived myoblasts and repair of damaged fibers^8^. SCs are located between the myofibre plasma membrane and sheathing basal lamina (BL)^9^, which provides a niche environment that is not thoroughly investigated. The muscle BL is a supra-molecular ECM structure connecting two networks of laminins and collagen polymers via the bridging function of glycoproteins and heparan sulfate proteoglycans, such as nidogen and perlecan^10^. Laminins belong to a family of sixteen distinct heterotrimer proteins made of one α, one β, and one γ subunit, and are critical for BL assembly and function^11^. The predominant laminin in healthy adult muscle fiber BL is a laminin-α2-containing isoform (laminin-211), although additional isoforms are present at the neuromuscular junction BL, and at the intramuscular nerve and vascular network BLs^12,13^. This adult muscle BL forms through the progressive replacement of the embryonic laminins, laminin-111 and laminin-511, by the adult isoform laminin-211 at the non-synaptic muscle BL during fetal and post-natal muscle development^13^. Interestingly, laminin-α5 has been reported to be upregulated transiently in human and mouse dystrophic muscle fiber BL, suggesting a degree of plasticity in the BL composition in the pathological muscle^12^. Given the role of laminin-111 in patterning and differentiation of skeletal muscle cells during embryonic development^14–16^, we investigated the role of the ‘embryonic’ laminin isoforms, laminins α1 and α5, in adult myogenesis.

Here, we report that upon SC activation, a remodeling event mediated by matrix metalloproteinases (MMPs) leads to the deposition of laminin-α1 and laminin-α5 at the SC niche during muscle regeneration. Notably, we observe a differential spatio-temporal distribution of laminin-α1 and laminin-α5 in the BL overlying activated SCs and regenerated myofibers, respectively. Loss-of-function of laminin-α1 impairs SC proliferation and self-renewal, and results in decreased long-term regenerative capability. Laminin-111 mediates its effects via integrin-α6β1 signaling, and by maintaining SC polarity and asymmetric cell division. Together, our observations indicate plasticity of the BL at the SC niche that supports SC propagation, differentiation, and self-renewal. These findings may be of importance for the design of therapeutic interventions for muscular dystrophies and to combat muscle aging.

## Results

### Laminin-α1 and laminin-α5 deposition at satellite cell niche

**Fig. 1:**
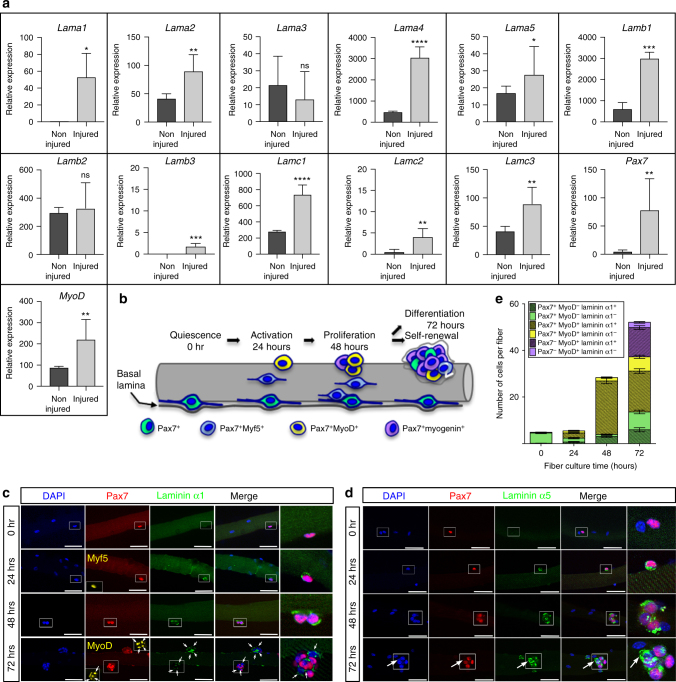
Laminin-α1 and laminin-α5 deposition in the niche of activated satellite cells. **a** Quantitative PCR analysis of *Lama1-5*, *Lamb1-3*, and *Lamc1-3* genes together with *Pax7* and *MyoD* in non-injured (dark gray; *n* = 3) and 4 dpi injured (light gray; *n* = 3) TA muscles. **b** Schematic representation of the myofiber culture system showing the sequential activation (blue), proliferation (yellow), self-renewal (green), and differentiation (purple) of satellite cells. **c** Representative immunofluorescence images of cultured myofibers analyzed for laminin-α1 (green), Pax7 (red), Myf5 (yellow at 24 h), and MyoD (yellow at 72 h). Panels on the right are high magnification images of the area boxed. White arrows indicate satellite cells associated with laminin-α1. Scale bar, 50 μm. **d** Immunofluorescence analysis of laminin-α5 (green) and Pax7 (red) distribution in cultured myofibers. Arrows indicate Laminin-α5 deposition in Pax7^−^cells at 72 h. Scale bar, 50 μm. **e** Quantification of the number of satellite cells expressing Pax7, MyoD, and laminin-α1 after myofiber culture. The color code relates to the cell populations described in (**b**). *n* = 3 experiments with 20–32 fibers analyzed per time point. Graph shows mean + sem; *P < 0.05, **P < 0.01, ***P < 0.001, ****P < 0.0001 (*t*-test)

To investigate whether muscle regeneration is associated with a degree of ECM remodeling, we used quantitative real-time PCR (qPCR) to determine the expression levels of all Laminin genes during murine skeletal muscle regeneration. *Tibialis anterior* (TA) muscle harvested at 4 days post cardiotoxin-mediated injury (dpi) was compared to non-injured TA muscle (Fig. 1a). *Pax7* and *MyoD* upregulation in injured TA samples confirmed the presence of muscle progenitor cells (Fig. 1a). In addition, our analysis showed significant increase of mRNA levels for *Lama1*, *Lama4*, *Lama5*, *Lamb1*, *Lamc1*, and moderate increase of mRNAs levels for *Lama2*, *Lamb3*, *Lamc2*, and *Lamc3* in 4 dpi injured TA muscle (Fig. 1a). Expression levels of *Lama3* and *Lamb2* were unchanged. Thus, muscle regeneration is accompanied by an upregulation of laminin-111 (α1β1γ1), laminin-411 (α4β1γ1), and laminin-511 (α5β1γ1). To confirm this findings, we used validated antibodies against laminin-α1^17,18^ and laminin-α5^19^ in an ex-vivo myofiber culture system in which SCs are retained in their niche in association with the myofiber and surrounding BL, and recapitulate the adult myogenic program over a 72-hour period^20^ (Fig. 1b). Pax7^+^ SCs did not express laminin-α1 and laminin-α5 in freshly isolated muscle fibers (0 h) (Fig. 1c–e). However, after 24 h in culture, laminin-α1 and laminin-α5 were detected at the SC niche exclusively, in contrast to laminin-α2 and laminin-β1, which were present throughout the BL overlying both myofiber and SCs (Fig. 1c–e and supplementary Fig. 1a). Interestingly, laminin-α1 deposition in the SC niche appeared transient starting with 53.1% of cells expressing at 24 h, peaking at 91.8% by 48 h before decreasing to 69.3% at 72 h. Laminin-α1 did not display a preferential association with particular fates and was associated with activated (Pax7^+^ Myf5^+^), proliferating (Pax7^+^ MyoD^+^), as well as differentiating (Pax7^−^MyoD^+^) and self-renewing (Pax7^+^ MyoD^−^) muscle progenitor cells. In contrast, laminin-α5 was preferentially associated with differentiating muscle progenitor cells (Fig. 1c–e and supplementary Fig. 1b).

**Fig. 2:**
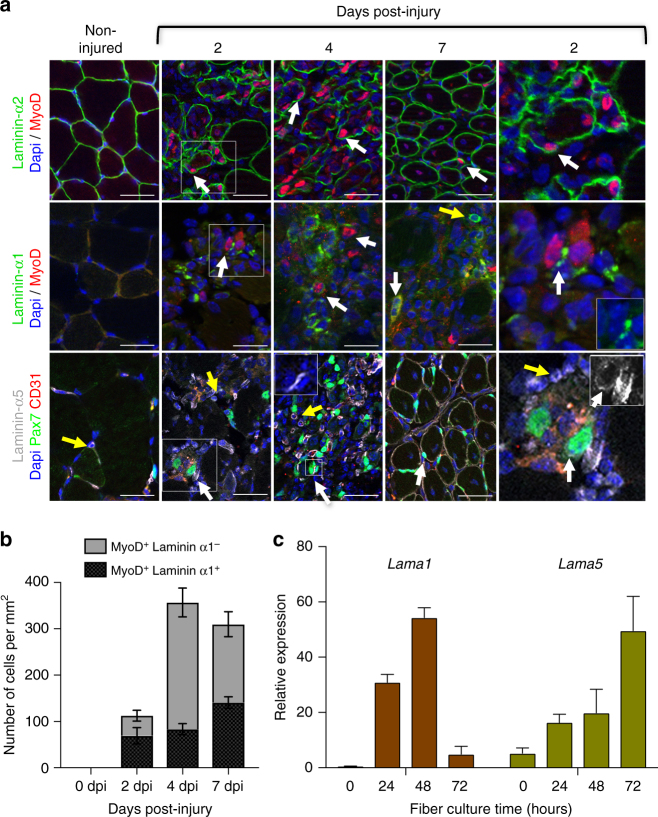
Spatio-temporal distribution of laminin-α1 and laminin-α5 in regenerating muscles. **a** Immunofluorescence analysis of laminin-α2 (green, top panels), laminin-α1 (green, middle panels), and laminin-α5 (gray, bottom panels) in regenerating TA muscle following cardiotoxin-mediated injury. Panels on the right are high magnification images of the 2 dpi images (indicated by a white rectangle, with small inserts showing the same image with the laminin channel only). The insert shown at 4 dpi in the laminin-α5 panels illustrates increased laminin α5 (white) deposition at the surface of satellite cells. White arrows indicate satellite cells. Yellow arrows indicate MyoD^−^ or Pax7^−^(GFP^−^) cells positive for laminin-α1 or α5. Scale bar, 50 μm. **b** Quantification of the number of laminin-α1^+^ myogenic cells in regenerating muscles. **c** qPCR analysis of *Lama1* and *Lama5* gene expression in myofibers cultured for 0–72 h. *n* = 6 experiments. Graphs show mean + sem

We next examined laminin-α1 and laminin-α5 deposition in the murine SC niche in vivo. We injected cardiotoxin into the TA muscle to cause widespread disruption of the muscle architecture. Regeneration of cardiotoxin-injured skeletal muscles typically span 14 days and is characterized by an intense inflammatory response between 1–7 dpi peaking at 4 dpi and a parallel SC-mediated repair program characterized by a large number of Pax7^+^ and MyoD^+^ activated SCs at 4 dpi, leading to the emergence of newly regenerated centrally-nucleated myofibers at 7 dpi (supplementary Fig. 1c-e). Consistent with previous reports^13,21,22^, laminin-α1 was not observed in non-injured adult muscles and laminin-α5 was confined to capillaries and blood vessels, and the neuromuscular junction (Fig. 2a and supplementary Fig. 1i,j). However, laminin-α1 was transiently observed in the niche of activated (MyoD^+^) SCs as early as 2 dpi, when it peaked at 61.5% of SC-derived progenitor cells, and remained associated with activated SCs until 7 dpi (Fig. 2a, b). By 14 dpi, laminin-α1 was no longer detected (Supplementary Fig. 1f). Interestingly, although laminin-α5 was also induced in SCs at 2 dpi, strong expression was not observed until 4 dpi, and by 7 dpi, laminin-α5 was distributed throughout the BL of regenerated, but not undamaged, muscle fibers (Fig. 2a and supplementary Fig. 1i). Thus, muscle regeneration is characterized by a dynamic change in the laminin composition at the extra-synaptic BL with the transient deposition of laminin-α1 at the activated SC niche and laminin-α5 at the BL of regenerated fibers. Laminin-α1 was also found associated with cells labeled with F4/80, a pan macrophage cell surface marker (supplementary Fig. 1g,h), suggesting that it may also contribute to the known cross-talk between SCs and macrophages^23^. Finally, laminin-α1 was also observed at the BL of activated SCs and macrophages in sedentary and exercised *mdx* mice, a model of human Duchenne muscular dystrophy (supplementary Fig. 2a-c). Laminin-α1 and laminin-α5 deposition into the SC and myofiber BL, respectively, is likely to result from the re-expression of these embryonic isoforms by SCs themselves or by myofibers since *Lama1* and *Lama5* transcripts peaked at 48 and 72 h in myofiber cultures, respectively, while no or little expression was observed in freshly isolated muscle fibers (Fig. 2c).

### Matrix metalloproteinases trigger remodeling of the SC niche

**Fig. 3:**
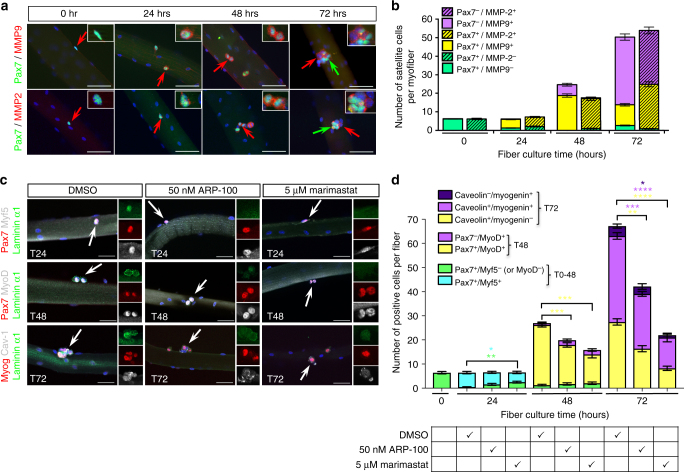
MMP2 and MMP9 catalyze SC basal lamina remodeling. **a** Immuno-localization of MMP2 and MMP9 (red) in EDL myofibers cultured for 72 h. Inserts are high magnification of satellite cells indicated by red arrows. Green arrows indicate Pax7^+^ satellite cells that are likely to be self-renewing cells and are negative for MMP2 and MMP9. Scale bar, 50 μm. **b** Quantification of the number of SCs expressing MMP2 and MMP9 in cultured EDL myofibers. The color code used relates to the cell populations described in Fig. 1b. *n* = 5 experiments with 50 myofibers per time point. **c** Representative images of control (DMSO) or treated myofibers cultured in the presence of the MMP inhibitors ARP-100 and Marimastat. Arrows indicate SCs shown at high magnification in inserts. Note the reduced deposition of laminin α1 after 24 h and 48 h in the presence of ARP-100 and the complete absence of laminin α1 after 24 h and 48 h in the presence of Marimastat. Scale bar, 50 μm. **d** Quantification of the effect of MMP inhibition by ARP-100 and Marismastat on the progression of SCs through the myogenic program in the ex-vivo myofiber culture system. *n* = 3 experiments with 25–40 fibers per time point. Graphs show mean + sem. *P < 0.05, **P < 0.01, ***P < 0.001, ****P < 0.0001 (*t*-test)

Laminin-α1 and laminin-α5 incorporation into the satellite cell BL soon after SC activation suggests an active mechanism to remodel locally the BL associated with SCs. Matrix metalloproteinases (MMPs) substrates include ECM components, and thus are good candidates to mediate BL remodeling during muscle regeneration^24,25^. We focused on the gelatinases MMP2 and MMP9, because gain and loss-of-function studies already suggested a link with skeletal muscle and the pathology of *mdx* mice^26–28^. Although no MMP2 or MMP9 expression was observed in freshly isolated myofibers, MMP2 and MMP9 were detected in activated SCs within 24 h in culture and remained associated with SCs undergoing expansion and differentiation (Fig. 3a, b). Muscle fibers from the Extensor Digitorum Longus (EDL) muscle were cultured in the presence of two different MMP inhibitors and assessed for the effect on laminin-α1 distribution and myogenesis. Consistent with the partial inhibition of laminin-α1 deposition into the SC niche, ARP-100, a MMP2-specific inhibitor^29^, had a significant, but transient effect on SCs proliferation (Pax7^+^MyoD^+^ at 48 h, Fig. 3c, d). Marimastat, a broad MMP inhibitor that blocks the activity of MMP1, 2, 7, 9, and 14^30^, clearly prevented laminin-α1 deposition in the SC niche, and caused a transient delay in SC activation (indicated by a higher number of Pax7^+^Myf5^−^ cells at 24 h) and a permanent decrease in the number of proliferating SCs (Pax7^+^MyoD^+^) at 48 h, and self-renewing (Caveolin^+^Myogenin^−^) and differentiating (Caveolin^+^Myogenin^+^) SCs at 72 h (Fig. 3c, d). Thus, MMP2 and MMP9 catalyze remodeling of the BL associated with activated SCs to facilitate laminin-α1 deposition, and inhibition of MMP-mediated BL remodeling impairs SC ability to progress through the myogenic program.

### Loss of laminin-α1 impairs SC proliferation and self-renewal

**Fig. 4:**
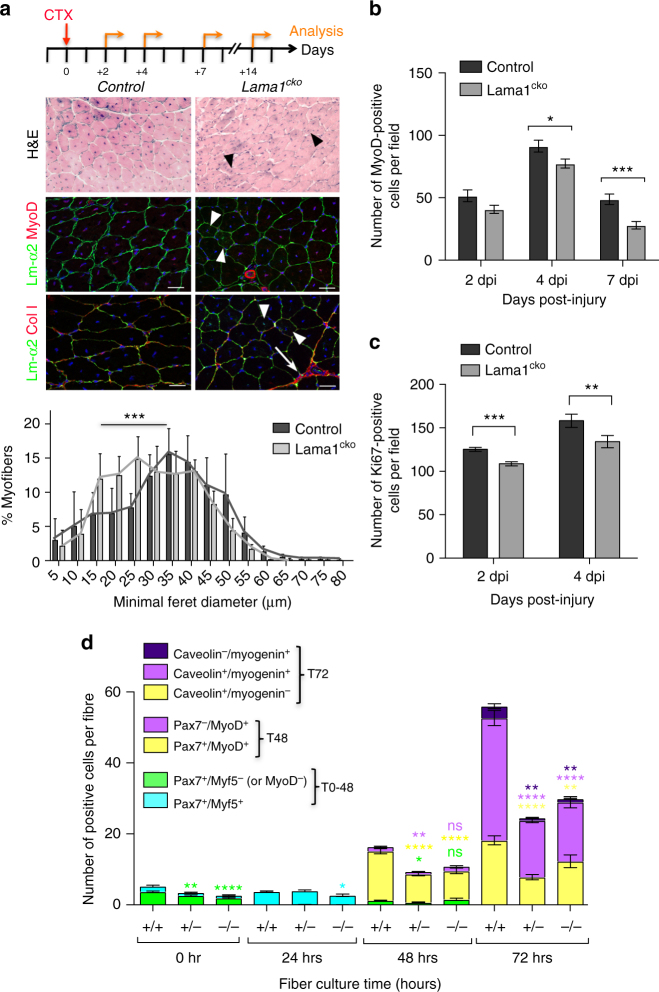
Laminin-α1 is essential for SC proliferation. **a** Control and *Lama1*^*cko*^ *Tibialis anterior* (TA) muscle analyzed at 14 days post injury by Haematoxylin and Eosin staining, and immunofluorescence for laminin-α2 (green), MyoD (red), and collagen I (red). Black and white arrowheads indicate the presence of smaller regenerated myofibers in *Lama1*^*cko*^ mice. White arrows indicate sites of fibrosis. The graph shows the minimal Feret diameter analysis of control and *Lama1*^*cko*^ TA muscles at 14 dpi. Scale bar, 50 μm. *n* = 3 per genotype. **b** Number of MyoD^+^ cells in injured TA muscles at 2, 4, and 7 dpi in control and *Lama1*^*cko*^ mice. *n* = 3 per genotype. **c** Number of Ki67^+^ cells in injured TA muscles at 2 and 4 dpi in control and *Lama1*^*cko*^ mice. **d** Quantification of the number of satellite cells activated (blue), proliferating (yellow), self-renewing (green), and differentiating (purple) in a 72-hour ex-vivo culture of control, heterozygous, and *Lama1*^*cko*^ myofibers. *n* = 3 per genotype with 31–79 myofibers per time point. Graphs show mean + sem. *P < 0.05, **P < 0.01, ***P < 0.001, ****P < 0.0001 (*t*-test for **b**, **c**, and **d**, and one-way ANOVA for **a**)

**Fig. 5:**
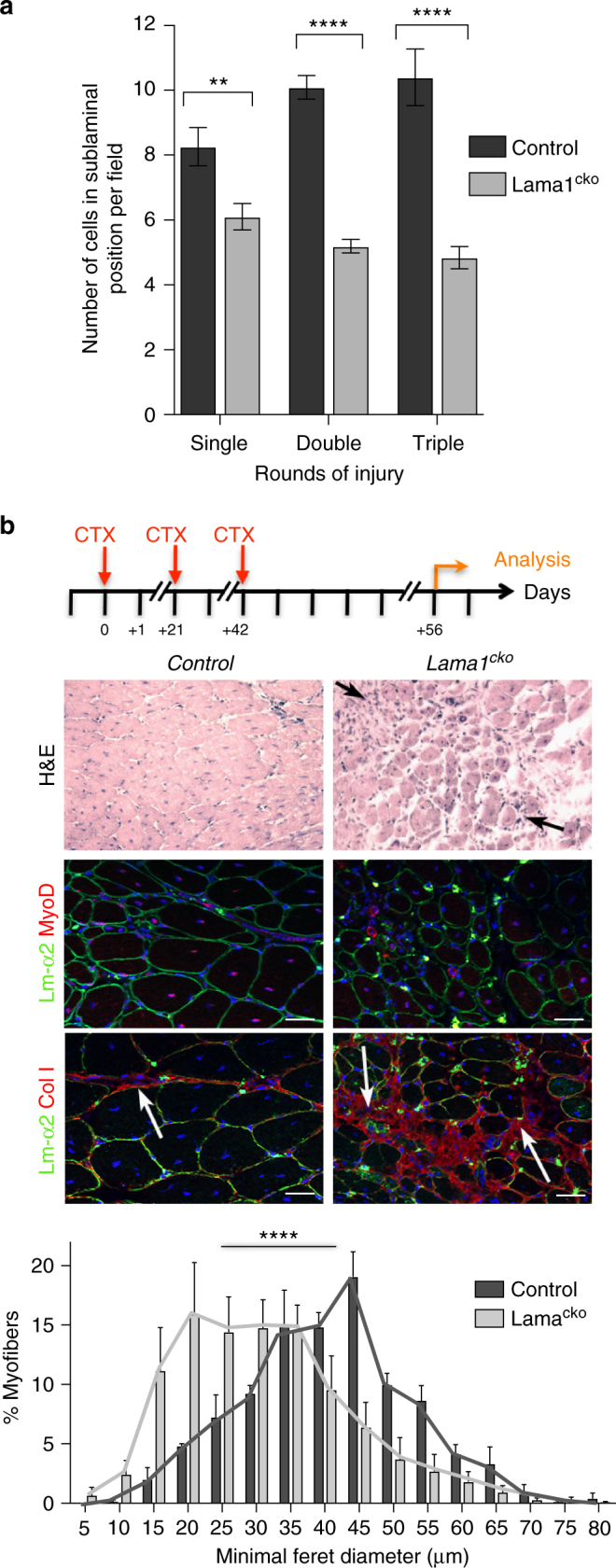
Impaired long-term regenerative capacity in *Lama1*-deficient mice. **a** Number of satellite cells returning to a sublaminal position in regenerated myofibers after one round (*n* = 3), two rounds (*n* = 3), and three rounds (*n* = 3) of injury. **b** Control and *Lama1*^*cko*^ mice analyzed at 14 dpi following three rounds of repeated injuries at 21-day intervals analyzed by Haematoxylin and Eosin staining and immunofluorescence for MyoD and collagen I (red). Black arrows indicate the presence of infiltrating inflammatory cells in *Lama1*^*cko*^ mice. White arrows indicate the site of fibrosis. The graph shows the minimal Feret diameter analysis of control and *Lama1*^*cko*^ mice after 3 rounds of injury. Scale bar, 50 μm. *n* = 6 (*Lama1*^*cko*^) and *n* = 3 (control). Graphs show mean + sem. *P < 0.05, **P < 0.01, ***P < 0.001, ****P < 0.0001 (*t*-test for a and one-way ANOVA for **b**)

To confirm that laminin-α1 deposition into the SC BL plays a role in SC activity and muscle regeneration, we examined a conditional knockout of *Lama1* (named *Lama1*^*cko*^ thereafter) generated by crossing *Sox2*^*Cre/+*^ and *Lama1*^*flox/+*^ mice^31,32^. As we previously reported^33^, *Lama1*^*cko*^ mice were born at normal mendelian ratios, although with a lower birth weight compared to their control littermate (*Lama*^*flox/flox*^), which recovered by 6 weeks of age (supplementary Fig. 3a). Muscle regeneration after cardiotoxin-mediated injury of the TA was not overtly affected in *Lama1*^*cko*^, although *Lama1*^*cko*^ regenerated myofibers were smaller than the control ones by 14 dpi (Fig. 4a and supplementary Fig. 3b). Consistent with this, there were fewer MyoD^+^ cells and Ki67^+^ cells in *Lama1*^*cko*^ mice at 2, 4, and 7 dpi, and fibers cultured from *Lama1*^*cko*^ mice had fewer proliferating SCs (Pax7^+^MyoD^+^) cells (Fig. 4b–d). Laminin-α5 was not upregulated in *Lama1*^*cko*^ mice (supplementary Fig. 3c), suggesting the absence of functional compensatory mechanisms that would explain the relatively mild phenotype observed. Notably, the numbers of self-renewing (Caveolin^+^Myogenin^-^) SCs (Fig. 4d) in *Lama1*^*cko*^ cultured myofibers and SCs returning to a sublaminal position at 14 dpi in *Lama1*^*cko*^ mice were reduced (Fig. 5a and supplementary Fig. 3d), suggesting a defect in SC self-renewal. To test this, we carried out three repeated injuries at 21-day intervals and analyzed animals after the 2^nd^ and 3^rd^ round of injury. Control mice repaired successfully their tissue 14 days after the second or third injury, indicating that a pool of SCs self-renewed and re-integrated into a sublaminal position after each injury (Fig. 5a,b). In contrast, *Lama1*^*cko*^ mice failed to regenerate their injured muscle, and displayed a high degree of fibrosis and the presence of MyoD^+^ cells, as well as a large number of infiltrating cells after the 3^rd^ round of injury (Fig. 5b), suggesting that SCs became depleted at each round of regeneration. Consistent with this observation, there were 26%, 48%, and 54% fewer self-renewing cells in *Lama1*^*cko*^ compared to control muscles after one, two, and three rounds of injuries, respectively (Fig. 5a). Together, these data demonstrate that in the absence of laminin-α1, SC fail to expand and self-renew efficiently during muscle regeneration, a defect that may originate in part from an earlier post-natal defect as suggested by the lower number of SCs present in *Lama1*^*cko*^ compared to control freshly isolated myofibers (Fig. 4d).

### Integrin-α6 mediates laminin-α1 signaling to SCs

**Fig. 6:**
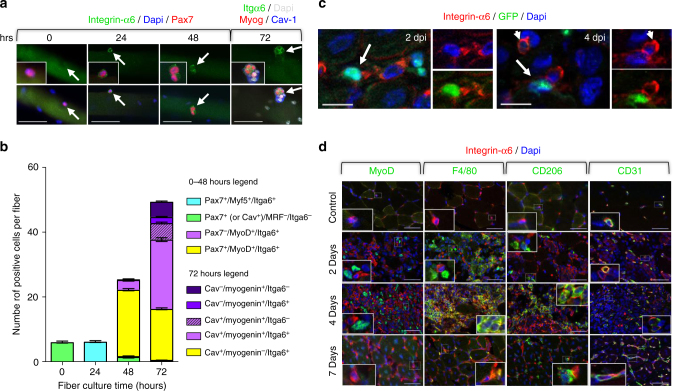
Integrin-α6 mediates laminin-α1 control of SC expansion. **a** Immunofluorescence analysis of cultured EDL myofibers showing integrin-α6 upregulation in activated SCs. Inserts show high magnification images of satellite cells indicated by the white arrows. Top panels show images in the green channel only, bottom panels show the merge images, inserts are higher magnification of merge image. Scale bar, 50 μm. **b** Quantification of integrin-α6 expression in satellite cells from cultured myofibers. *n* = 3 with 20–30 fibers per time point. **c** Integrin-α6 (red) distribution in 2 dpi and 4 dpi injured TA muscle of Tg(Pax7-GFP) mice. Images of the blue/red channels and green/red channels show colocalization of integrin-α6 with satellite cells (white arrows) and with predicted endothelial cells (white arrowheads). Scale bar, 20 μm. **d** Immuno-colocalization of integrin-α6 (red) with either MyoD (green, activated satellite cell marker), F4/80 (green, pan macrophage marker), CD206 (green, M2 macrophage marker), or CD31 (green, endothelial cell marker) following cardiotoxin-mediated injury of TA muscles. Inserts are high magnification images of areas delineated by a dotted white line. Note that integrin-α6 is primarily associated with endothelial cells at 2 dpi, whereas it is primarily detected at the surface of macrophages (M1 and M2) and satellite cells at 4 dpi. By 7 dpi, integrin-α6 returns to being restricted to endothelial cells. Scale bar, 50 μm. Graph shows mean + sem

**Fig. 7:**
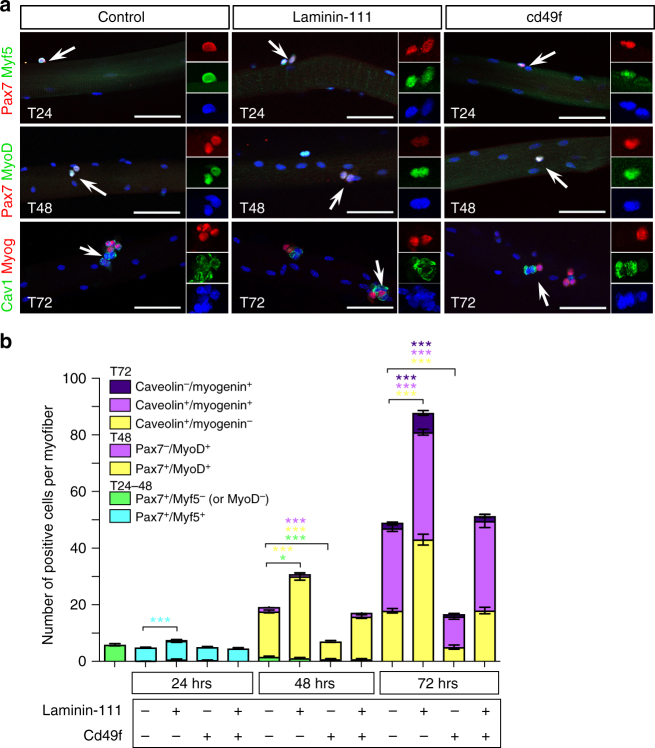
Laminin-111 treatment increases SC proliferation and self-renewal. **a** Representative images of myofibers cultured in control conditions or in the presence of laminin-111 or Cd49f, and analyzed by immunofluorescence for Pax7 (red), Myf5 (green), MyoD (green), Caveolin-1 (green), and Myogenin (red). Scale bar: 50 μm. **b** Quantification of satellite cell populations in myofiber cultures supplemented with Laminin-111, Cd49f, or both. Graph shows mean + sem. *P < 0.05, **P < 0.01, ***P < 0.001 (*t*-test)

The deposition of laminin-α1 (presumably laminin-111) at the SC niche may confer specific properties to the BL that support SCs self-renewal and progression through myogenesis. We hypothesized that this change in laminin composition initiates distinct signaling in SCs. Indeed, although laminin-α1 can bind to integrin-α7β1, the laminin-α2 receptor normally expressed in adult muscles^34^, it binds preferentially to integrin-α6β1, a receptor highly expressed in embryonic muscle^16^. Thus, we asked whether integrin-α6 was also re-expressed during adult myogenesis. No integrin-α6 was detected in freshly isolated myofibers (Fig. 6a). However, integrin-α6 was upregulated in activated SCs at 24 h and remained expressed in SCs at 48 and 72 h, albeit at lower levels (Fig. 6a, b). Integrin-α6 was also dramatically, but transiently, upregulated in a sequential manner in vivo in endothelial cells (CD31^+^), M1 and M2 macrophages (F4/80^+^ and CD206^+^), and in SCs (Pax7^+^ and MyoD^+^) following cardiotoxin-mediated muscle injury, an observation that corroborates previous reports^19^ (Fig. 6c, d). Preventing signaling through integrin-α6 with cd49f, an integrin-α6 blocking antibody, impaired SC expansion in a manner reminiscent of the defect observed in myofiber cultures from *Lama1*^*cko*^ muscles (compare Fig. 7a, b to Fig. 4d). Notably, there was a significant reduction of the number of Caveolin-1^+^/Myogenin^-^ cells at 72 h, which comprises self-renewing SCs (Fig. 7b). Conversely, culturing fibers in the presence of exogenous laminin-111 resulted in a 2-fold increase in the number of SCs by 72 h, with a greater effect on the population of Caveolin-1^+^/Myogenin^-^ cells deemed to self-renew (Fig. 7a, b). When muscle fibers were cultured in the presence of both laminin-111 and Cd49f, no increase in SC number was observed (Fig. 7a, b), confirming that laminin-111 effects on SCs are mediated through integrin-α6.

### Laminin-111 treatment promotes symmetric cell division

**Fig. 8:**
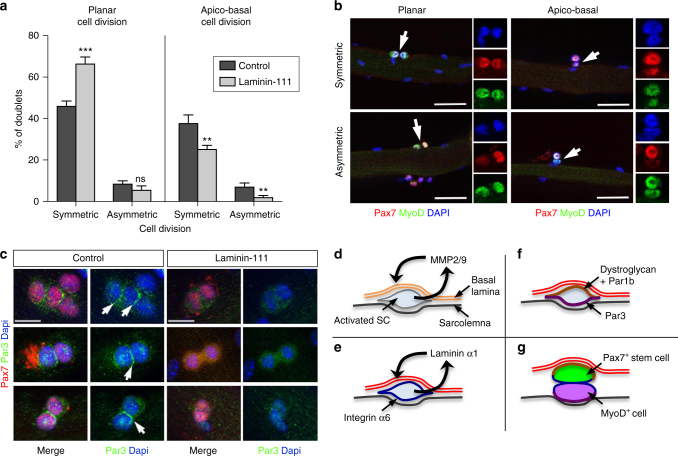
Laminin-111 treatment alters SC cell polarity and stimulates planar cell division. **a** Quantification of planar and apico-basal cell divisions (symmetric and asymmetric) based on the expression of Pax7 and MyoD in T46 myofibers treated with PBS (control; dark gray) or laminin-111 (light gray). *n* = 3 with 50–82 doublets analyzed per culture. *P < 0.05, **P < 0.01, ***P < 0.001 (*t*-test). **b** Representative immunofluorescence images of planar and apico-basal cell divisions in T46 myofibers analyzed using antibodies against Pax7 (red) and MyoD (green). White arrows indicate cell doublets. Individual color channels are shown. Scale bar: 50 μm **c** Representative immunofluorescence of Par3 (green) and Pax7 (red) in myofibers cultured for 46 h in in the presence of PBS (control) or laminin-111. Arrows indicate Par3 asymmetric distribution in control, but not in laminin-111 treated fibers. The white star indicates background staining. Scale bar: 10 μm. **d**–**g** Proposed model for laminin-111 control of SC self-renewal: **d** Upon activation, SCs upregulate MMP2 and MMP9 expression, leading to a local digestion of the laminin-α2-containing basal lamina (double orange line) at the SC niche. **e** Simultaneously, SCs re-express laminin-α1, which is secreted and deposited into the SC basal lamina (double red line), and the laminin-α1 receptor, integrin-α6 (blue line). **f** Laminin-α1-mediated signaling initiates or maintains SC polarity through the asymmetric distribution of the basal determinant Par1b (brown) and the apical determinant Par3 (purple), leading to apico-basal cell polarity. **g** Apico-basal asymmetric cell division yields two distinct daughter cells, including a self-renewing SC associated with the basal lamina (Pax7^+^ in green) and a differentiating SC (MyoD^+^ in purple)

SCs can divide either in a planar orientation where both daughter cells are in contact with the fiber and the BL or in an apico-basal orientation where one daughter cell in contact with the BL adopts a self-renewal cell fate (Pax7^+^) and the daughter cell in contact with the myofiber adopts a myogenic cell fate (MyoD^+^)^35,36^. To address how laminin-111 treatment causes an increase in the number of self-renewing cells, we monitored planar and apico-basal cell divisions and recorded whether daughter cells in doublets were identical (symmetric cell division) or distinct (asymmetric cell division) in myofibres cultured for 46 h. Laminin-111 treatment caused an increase in the proportion of cell doublets dividing in a planar symmetrical orientation, at the expense of cells dividing in an apico-basal orientation (Fig. 8a, b). Apico-basal cell division is linked to the asymmetrical distribution of cell polarity proteins Par1 and Par3^36,37^. We observed that Par3 was indeed asymmetrically distributed in dividing progenitor cells, some of which had downregulated Pax7 and were fated to differentiate, in control conditions (Fig. 8c). In laminin-111-treated myofibers, Par3 was downregulated or uniformly expressed at low levels (Fig. 8c). Thus, the addition of laminin-111 may interfere with the normal distribution of cell polarity proteins and cause cells to divide in a planar orientation and adopt a stem cell fate.

## Discussion

Adult organ homeostasis relies on the activity of tissue-specific stem cells, which are controlled by extrinsic factors supplied by a highly specific micro-environment known as the 'stem cell niche’^1,38^. Studies of the past decade have greatly contributed to our current understanding of the stem cell niche characteristics, in particular in germline stem cells of *Drosophila*, and in haematopoietic, hair follicle and intestinal crypt stem cells in mammals^1^. SCs operate also within a niche environment, which provides cues for SC activation, proliferation, differentiation, and self-renewal^39,40^. Our results demonstrate that upon activation, SCs pro-actively modify the ECM within their niche by producing enzymes that catalyze the remodeling of the SC BL and by synthesizing sequentially laminin-α1 and laminin-α5, two laminin subunits that are normally associated with embryonic myogenesis^14^. The deposition of laminin-111 into the SC BL is essential for SC expansion and self-renewal, a process mediated by the signaling of laminin-α1 through integrin-α6. Thus, remodeling at the SC BL is a novel mechanism that contributes to the control of SC self-renewal^36,41–43^. Two recent reports have implicated the ECM molecules collagen type VI and fibronectin in the control of SC activity and self-renewal^44,45^, suggesting that remodeling of the ECM following muscle injury and SC activation is a widespread mechanism to maintain a stem cell pool. However, it is worth noting that while laminin-111 associates directly with SCs via its receptors, both collagen VI and fibronectin assemble into fibrillar networks that are components of the interstitial matrix often associated with fibroblasts. Furthermore, the broad upregulation of fibronectin and collagen VI in injured muscles, reminiscent of what we observed with laminin-α5, contrasts with the local upregulation of laminin-α1 reported in this study, and suggests that the mechanisms underlying laminin-α1 function in SC self-renewal differ from those of fibronectin and collagen VI. Consistent with this, while fibronectin binds to syndecan 4 and modulates frizzled-mediated Wnt response to maintain SCs in their niche^43^, laminin-α1 mediates its effect on SC self-renewal through integrin-α6β1. Given that SC self-renewal is maintained through asymmetric cell division and that SC asymmetric cell division occurs primarily following apico-basal cell division, which yields a daughter stem cell associated with the BL and a daughter cell committed to differentiation^35^, one may speculate that laminin-α1 deposition in the SC niche acts as an initiating event in the process of asymmetric cell division. Interestingly, dystroglycan, another laminin receptor and component of the dystrophin-associated glycoprotein complex, was recently shown to be asymmetrically distributed and to associate with Par1b, a microtubule-associated cell polarity protein, in SCs fated to self-renew following apico-basal cell division^37^. Here, we showed that exogenous laminin-111 interferes with the apico-basal distribution of Par3 and promotes planar cell division at the expense of apico-basal cell division. This suggests a possible mechanism whereby upon activation SC-mediated deposition of laminin-111 into the SC BL initiates a cascade of events leading to clustering of dystroglycan on the basal side, as we previously observed in the myotomal basement membrane in the embryo^46^, the apico-basal localization of Par1b and Par3, and apico-basal cell division to produce two daughter cells with distinct fates (Fig. 8d–f).

Our data further suggest that laminin-111 mediate its effects on SCs via integrin α6β1. Consistent with a possible involvement of integrin-α6β1 in laminin-111-mediated SC self-renewal, Collins-Hooper et al. observed higher levels of integrin-α6 in SCs from young compared to old muscles^47^, suggesting that loss of self-renewal capability observed in aged mice^48^ may be partially due to reduced integrin-α6β1 signaling. Given that the axis laminin-111/integrin-α6β1 has been associated with long-term self-renewal of induced-pluripotent stem cells^49,50^, with sphere-forming capacity of human prostate cancer stem cells and neural stem cells^51,52^, and with asymmetric cell division of *Drosophila* ovarian follicle stem cells^53^, it is likely that interaction between laminins and integrins represents an ancient mechanism to maintain stem cell self-renewal. Further investigations are required to uncover the downstream processes controlled by this signaling pathway.

The implications of this study to regenerative medicine are considerable. Indeed, congenital muscular dystrophies (CMD) are a group of devastating degenerative diseases of the skeletal muscular tissue caused by mutations in proteins involved directly or indirectly in the association between the muscle fiber and the ECM^54^. There is currently no therapy for this group of diseases, although recent studies reported that overexpression of laminin-α1 or injection of laminin-111 improved dramatically the dystrophic phenotype in mouse models of CMD^55–57^. Laminin-111 is thought to rescue the dystrophic phenotype by stabilizing the sarcolemma and preventing contraction-induced damage of CMD muscles. Our findings reveal that exogenous laminin-111 is also likely to improve the dystrophic phenotype of CMD models by augmenting SC expansion and self-renewal. This is consistent with previous studies^58^, showing that SCs cultured on laminin-111 support have a higher regenerative capability than SCs cultured on fibronectin when engrafted in *mdx* dystrophic muscles. Together with our findings, this provides strong support for the use of laminin-111 in approaches to generate SCs or induced-pluripotent stem cells-derived SCs for cell therapy.

## Methods

### Mice

All mice were housed in the temperature and humidity-controlled barrier facility of the University of Sheffield. Experimental procedures were performed in accordance with the Animals (Scientific Procedures) Act 1986, were approved by the University of Sheffield Ethical Review Process committee, and performed under UK Home Office Project Licence 60/4354. All experiments were performed on mice at 8–12 weeks of age. C57BL/6, *mdx* (kindly provided by Gaynor Miller), *Lama1*^*flox/*+30^, Tg(*Sox2-cre*)^1A*mc*^ (kindly provided by Elizabeth Robertson)^31^, and Tg(Pax7-GFP) (kindly provided by Shahragim Tajbakhsh)^5^ were maintained on a C57BL/6 background in accordance to the Home Office guidelines for animal handling and care. Exercised C57BL/6 (control) and *mdx* mice were allowed access to voluntary wheel running exercise from the age of 4 weeks for a period of 17 days. The average distance ran per day was recorded on a pedometer and was 3.23 km/day ± 0.41 for *mdx* mice and 4.02 km/day ± 1.21 for C57BL/6 mice. Primers and protocols used for genotyping of the mice are listed in Supplementary Table 1.

### Muscle injury

Muscle injury was induced on 8-week-old mice by a single 50 μl injection of 10 μM Cardiotoxin (CTX) from *Naja mossambica* (Latoxan) into the left TA muscle. At various times following injury (2, 4, 7, and 14 days), the mice were culled, injured, and contralateral control muscles were harvested for analysis. Transverse sections of muscles were subjected to Haematoxylin-Eosin staining to confirm the degree of injury and regeneration. The minimal Ferret’s diameter of fibers was determined on transverse sections immuno-labeled with laminin-α2 using the ImageJ software.

### EDL muscle fiber culture

EDL muscles were dissected from 6–8-week-old C57BL/6 or *Lama1*^*cko*^ mice and incubated for 60–90 min at 37 °C in 0.2% Collagenase type I (2 mg/ml, Sigma) freshly prepared in DMEM + Glutamax medium with 1% PSF (antibiotic/antimycotic solution, Sigma). The muscles were then transferred to DMEM medium supplemented with 10% horse serum (Invitrogen), 0.5% chick embryo extract (Seralab), and 1% PSF, and the myofibres were harvested by gentle flushing of medium using a flamed-polished glass Pasteur pipette. Single myofibres were washed and transferred to a new Petri dish, and either fixed immediately in 4% paraformaldehyde (PFA) for 6 min (time 0 h) or cultured for up to 72 h in tissue culture dishes (Nunclon) coated with 5% bovine serum albumin (BSA, Sigma) at 37 °C in 5% CO_2_. Where indicated, MMP inhibitors, ARP-100 (50 nM, Alfa Aesar J64151) or Marimastat (5 μM, R&D systems), integrin-α6 blocking antibody Cd49f (30 μg/ml, clone GoH3 MCA699, AbD Serotec), or soluble laminin-111 (30 μg/ml, Millipore CC095) were added to the medium. At the end of culture time, myofibres were fixed in 4% PFA for 6 min if processed for immunofluorescence or directly transferred to Trizol (Life Technologies) if processed for qPCR.

### Immunofluorescence

Fixed myofibres were washed in phosphate buffer saline (PBS, Fisher Scientific) and permeabilized in 0.5% Triton X100 (Sigma) for 8 min. Skeletal muscles were harvested, fixed in 4% or 2% PFA for 2 h at 4 °C, and washed twice in PBS. Skeletal muscles were then transferred into 20% sucrose in PBS overnight at 4 °C followed by 3–4 h in 30% sucrose. Finally, the muscles were dipped in OCT (VWR) and immediately frozen in liquid nitrogen-cooled Isopentane (VWR). A total of 7 µm cryosections were collected on superfrost slides (Menzel-Glaser) using a cryostat (Bright Instruments). Blocking was performed in 20% horse serum in PBS (for isolated muscle fibers) or in blocking solution (5% BSA, 2% heat-inactivated goat serum, 2% fetal bovine serum (FBS), 0.05% Triton X100 in PBS for muscle cryosections) for 1 h at room temperature. Appropriate primary antibody diluted in PBS (for myofibres) or in PHT (1% heat-inactivated goat serum, 0.05% Triton X100 in PBS for cryosections) was added and incubated overnight at 4 °C. After three washes in 0.05% Triton X100 in PBS (for myofibres) or in PHT (for cryosections), the secondary antibody diluted in PBS or PHT was added and incubated for 1 h at room temperature. After three washes, the myofibres were transferred on slides and mounted in Vectashield with DAPI (Vector labs). Where two antibodies raised in the same species were used, primary and secondary antibody detection using the first antibody was carried out as described above, followed by an incubation with normal serum from the host species of the primary antibody prior to detection using the second conjugated antibody. Primary antibodies used were anti-caveolin-1 (1:400; sc-894, Santa Cruz), anti-pax7 (1:20, DHSB), anti-myoD (1:1000; sc-304, Santa Cruz), anti-myf5 (1:2000; sc-302, Santa Cruz), anti-myogenin (1:50; F5D, DHSB), anti-laminin-α2 (1:200; 4H8–2, Enzo), anti-laminin-α1 (MAB-1903 at 1:200, Chemicon; mab200 at 1:2, and sc-65645 at 1:100, Santa Cruz), anti-laminin-α5 (1:10000; clone 405, a gift from L. Sorokin^59^), anti-laminin-α1 (1:200; MAB1905, Chemicon), anti-integrin-α6 (1:40; MCA699, AbD SeroTec), FITC anti-F4/80 (1:100; ab105155, AbCAM), FITC anti-CD206 (1:250; clone C068C2, BioLegend UK), anti-CD31 (1:100; AF3628, R&D Systems), anti-Collagen I (1:300; AB765P, Chemicon), anti-Ki67 (1:300; NCL-Ki67p, Novocastra), anti-MMP9 (1:200; sc-6841, Santa Cruz), anti-MMP2 (1: 300; sc-10736, Santa Cruz), and anti-Par3 (1:750; 07–330, Millipore). Secondary antibodies were Alexa 488 goat anti-rabbit IgG (A11034), donkey anti-rabbit IgG (A21206), donkey anti-mouse IgG (A21202), and donkey anti-rat IgG (A21208) or Alexa 594 goat anti-rabbit IgG (A11037), donkey anti-goat IgG (A11058), and goat anti-mouse IgG (A11005) (all used at 1:500, Molecular Probes). To record cell divisions, only doublets were considered. The following criteria were used: planar cell divisions were parallel to the myofiber axis, apico-basal cell divisions were perpendicular to the fiber axis, symmetrical cell divisions included daughter cells with same immunolabeling (Pax7^+^MyoD^+^), asymmetrical cell divisions included two daughter cells with distinct immunolabeling (for instance, Pax7^−^MyoD^+^ and Pax7^+^MyoD^+^). Images were captured on a Zeiss Apotome microscope using the Axiovision imaging system. The images were assembled using Photoshop CS version 6.

### Quantitative PCR

Total RNA was isolated using Trizol (Invitrogen) according to the manufacturer protocol, and cDNAs were synthesized using the Superscript III First Strand Synthesis System using random hexamers (Invitrogen). qPCR was carried out on an iCycler instrument (Biorad) using the SYBR green reagents (Sigma) or a StepOne real-time PCR instrument (Applied Biosystems) using TaqMan reagents (Applied Biosystems). The cycling conditions used were as follows: iCycler: 10 min at 95 °C, and 40 cycles including a 15 s denaturation at 94 °C, 10 s annealing at the primer Tm°C, and a 15 s extension at 72 °C; StepOne: a 20 s denaturation at 95 °C, followed by 40 cycles of 95 °C for 1 s and 60 °C for 20 s. Transcript levels were normalized to glyceraldehyde 3-phosphate dehydrogenase (GAPDH) transcript levels (iCycler) or to eukaryotic 18 s rRNA (Thermo Fisher Scientific). Primers used are described in Supplementary Table 2. Applied Biosystems StepOne Software V2.3 was used to analyze the data and relative expression levels were calculated using the 2^−ΔCT^ method^60^.

### Statistical analyses

Data are presented as the mean ± standard error of the mean (s.e.m). A minimum of three myofibre cultures with on average 10–20 myofibres per culture or three mice per genotype and time point with 6–10 sections per muscle was analyzed. Statistical analyses were performed on the mean values from different experiments (myofiber cultures or mice). Comparisons between groups used unpaired *t*-test assuming two-tailed distributions or one-way ANOVA using GraphPad Prism 7 for Macintosh (http://www.graphpad.com).

### Data availability

The authors declare that all data supporting the findings of this study are available within the article and its Supplementary Information files or from the corresponding author upon reasonable request.

## Electronic supplementary material

Supplementary Information
