# Supplementary material for: Basal lamina remodeling at the skeletal muscle stem cell niche mediates stem cell self-renewal
Source: Nat Commun. 2018 Mar 14;9:1075. doi: 10.1038/s41467-018-03425-3 (PMC5852002; doi:10.1038/s41467-018-03425-3)
Supplement: Supplementary file 1 — Supplementary Information [file 41467_2018_3425_MOESM1_ESM.pdf]

Basal lamina remodeling at the skeletal muscle stem cell niche mediates stem cell self-renewal.

Rayagiri et al.

Supplementary information

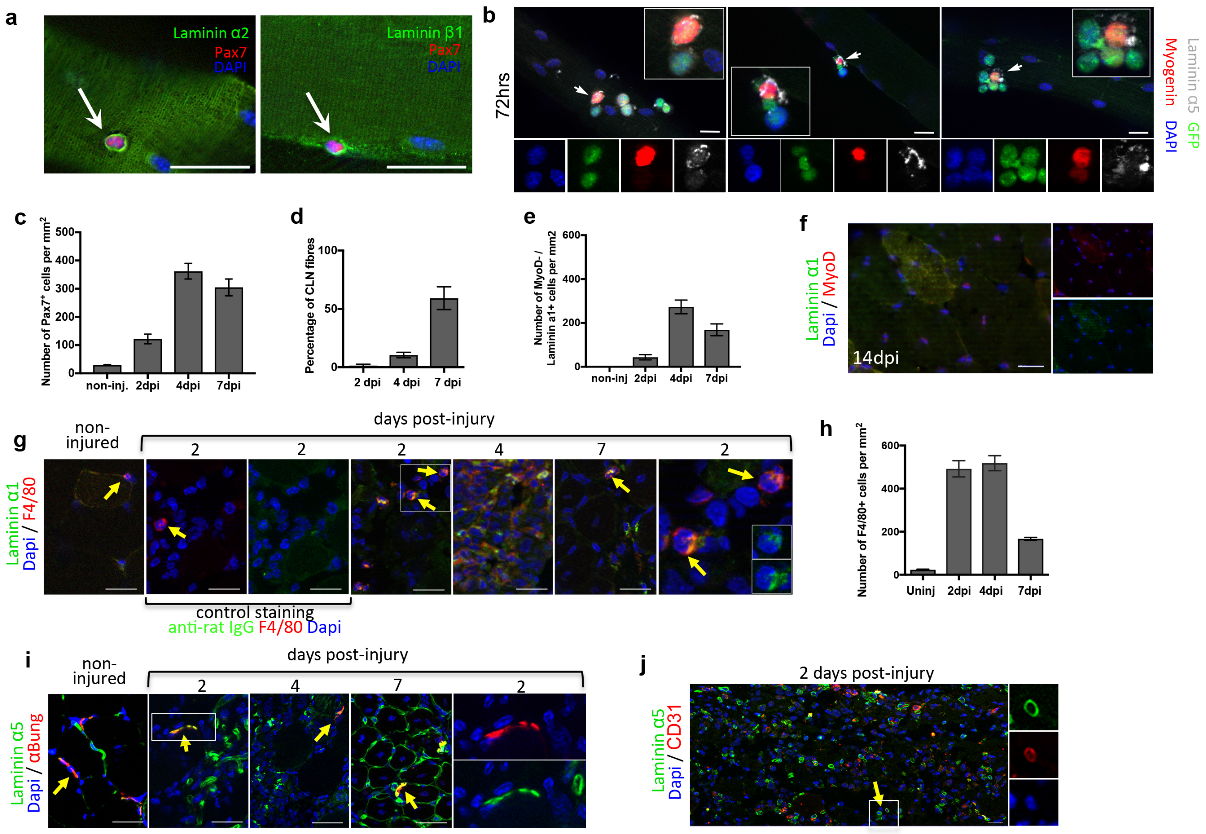

Supplementary Figure 1: Laminin-α1 and laminin-α5 duing muscle regeneration following cardiotoxin-mediated injury. (a) Laminin-α2 and laminin-β1 are uniformly detected within the muscle basal lamina with higher expression at the satellite cell niche. Immunofluorescence analysis of freshly isolated EDL muscle fibers with antibodies against Pax7 (red) and laminin-β1 or α2 (green). White arrows indicate satellite cells. Scale bar, 30μm. (b) Laminin-α5 associates preferentially with differentiating muscle progenitor cells. Immunofluorescence analysis of 72-hour fiber cultures from Tg(Pax7-GFP) EDL muscles with antibodies against GFP (green), Myogenin (red) and laminin-α5 (white). Nuclei are counterstain with DAPI (blue). Three distinct examples are shown with separate channels displayed underneath the main image. An enlarged merged view is shown as insert. White arrows show the selective presence of laminin-α5 at the surface of Myogenin-positive cells. Scale bar: 20μm. (c) Quantification of the number of $Pax7^+$ cells per mm^2 immuno-detected on transverse sections of TA muscle at 2, 4, and 7dpi following cardiotoxin-

mediated injury. (d) Percentage of fibers containing centrally-located nuclei on transverse sections of TA muscle at 2, 4, and 7dpi following cardiotoxin-mediated injury. (e) Quantification of the number of laminin-α1-positive/MyoD-negative cells per mm^2 on transverse sections of TA muscle at 2, 4, and 7 following cardiotoxin-mediated injury. (f) Transverse section of 14dpi TA muscle analyzed by immunofluorescence using antibodies against laminin-α1 (green) and MyoD (red). Note the down-regulation of laminin-α1. Scale bar: 50µm. (g) Representative images of transverse sections of C57BL/6 TA muscle following cardiotoxin-mediated injury analyzed by immunofluorescence with antibodies against laminin-α1 (green) and F4/80 (red). Right panel shows a higher magnification image of insert indicated by white rectangle at 2dpi (with green channel in insert). Yellow arrows show the co-localization of laminin-α1 and F4/80 in macrophages, except in the panels labeled 'control staining' showing the absence of non-specific staining using an Alexa Fluor 594 anti-rat IgG in F4/80 labeled tissue sections (indicated by the yellow arrow). Scale bar: 50µm. (h) Quantification of the number of F4/80-positive cells per mm^2 on transverse sections of TA muscle at 2, 4, and 7 following cardiotoxin-mediated injury. (i) Representative images of transverse sections of C57BL/6 TA muscle following cardiotoxin-mediated injury analyzed by immunofluorescence with antibodies against laminin-α5 (green) and conjugated α-Bungarotoxin (red). Right panels show higher magnification images in red and green channels of insert indicated by white rectangle at 2dpi. Yellow arrows show the co-localization of laminin-α5 and α-Bungarotoxin at the neuro-muscular junction. Scale bar: 50µm. (j) Representative image of 2dpi TA muscle analyzed by immunofluorescence with antibodies against laminin-α5 (green) and CD31 (red). Right panels show higher magnification images in red, green, and blue channels of insert indicated by white rectangle. Yellow arrows show the co-localization of laminin-α5 and CD31 in endothelial cells. Scale bar: 50µm.

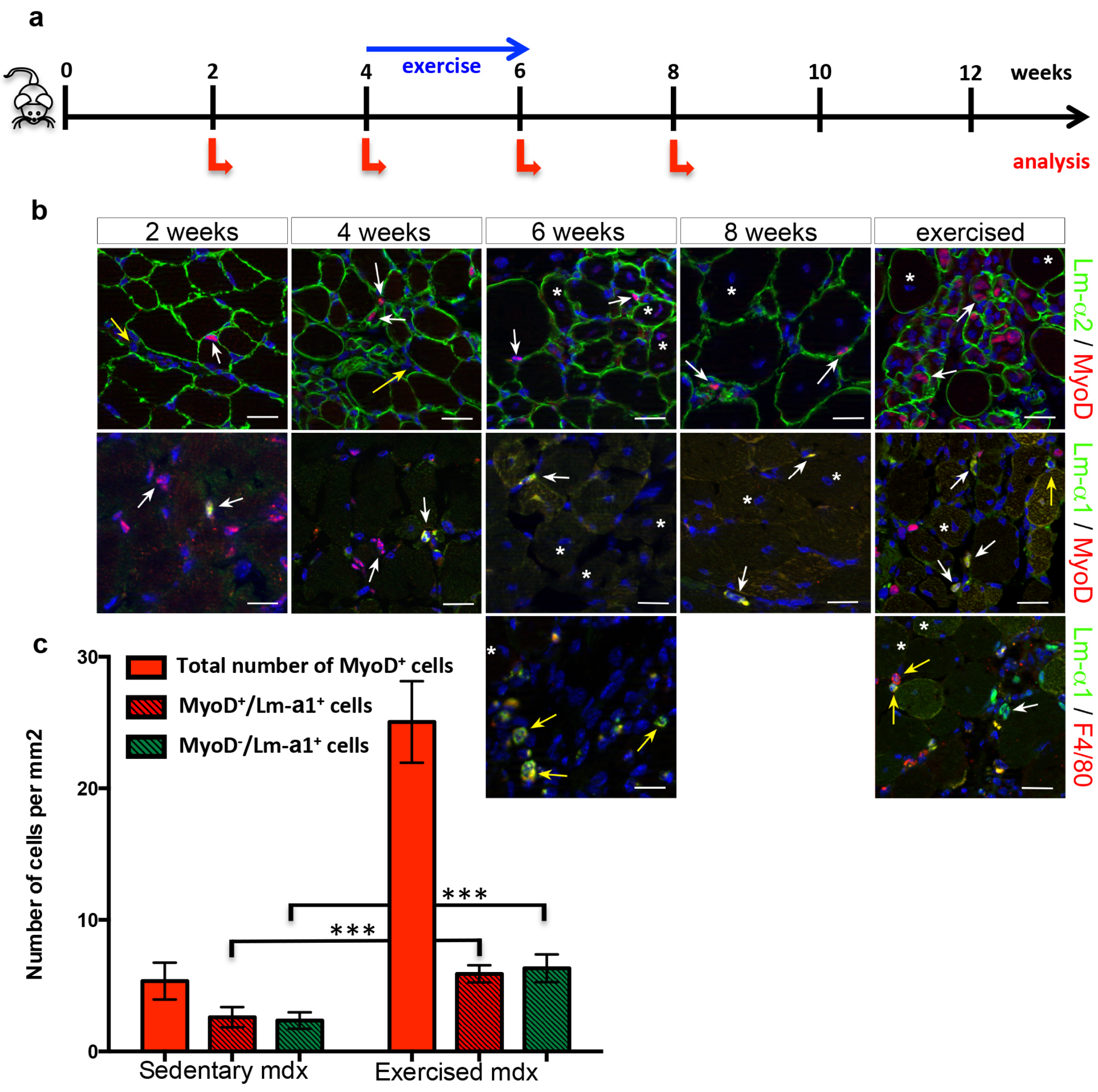

Supplementary Figure 2: Laminin α1 deposition in the SC basal lamina in regenerating *mdx* muscles. (a) Schematic representation of the analysis of sedentary and exercised *mdx* mice. (b) Representative images of transverse sections of EDL muscles from 2, 4, 6 and 8-week old sedentary *mdx* mice and from 8-week old exercised mdx mice to detect laminin-α1 (green) expression at the surface of satellite cells (MyoD, red) and macrophages (F4/80, red). White arrows indicate activated satellite cells and yellow arrows indicate inflammatory cells, including macrophages. Scale bar: 50µm. n=3 animals per time point (c) Number of laminin-α1-expressing cells per mm^2 in 8-week old sedentary and exercised *mdx* EDL muscles. Red hatched bars show $MyoD^+$ satellite cells and green hatched bars show $MyoD^-$ macrophages. Graphs show mean + sem. ****P* < 0.001 (one-way ANOVA test).

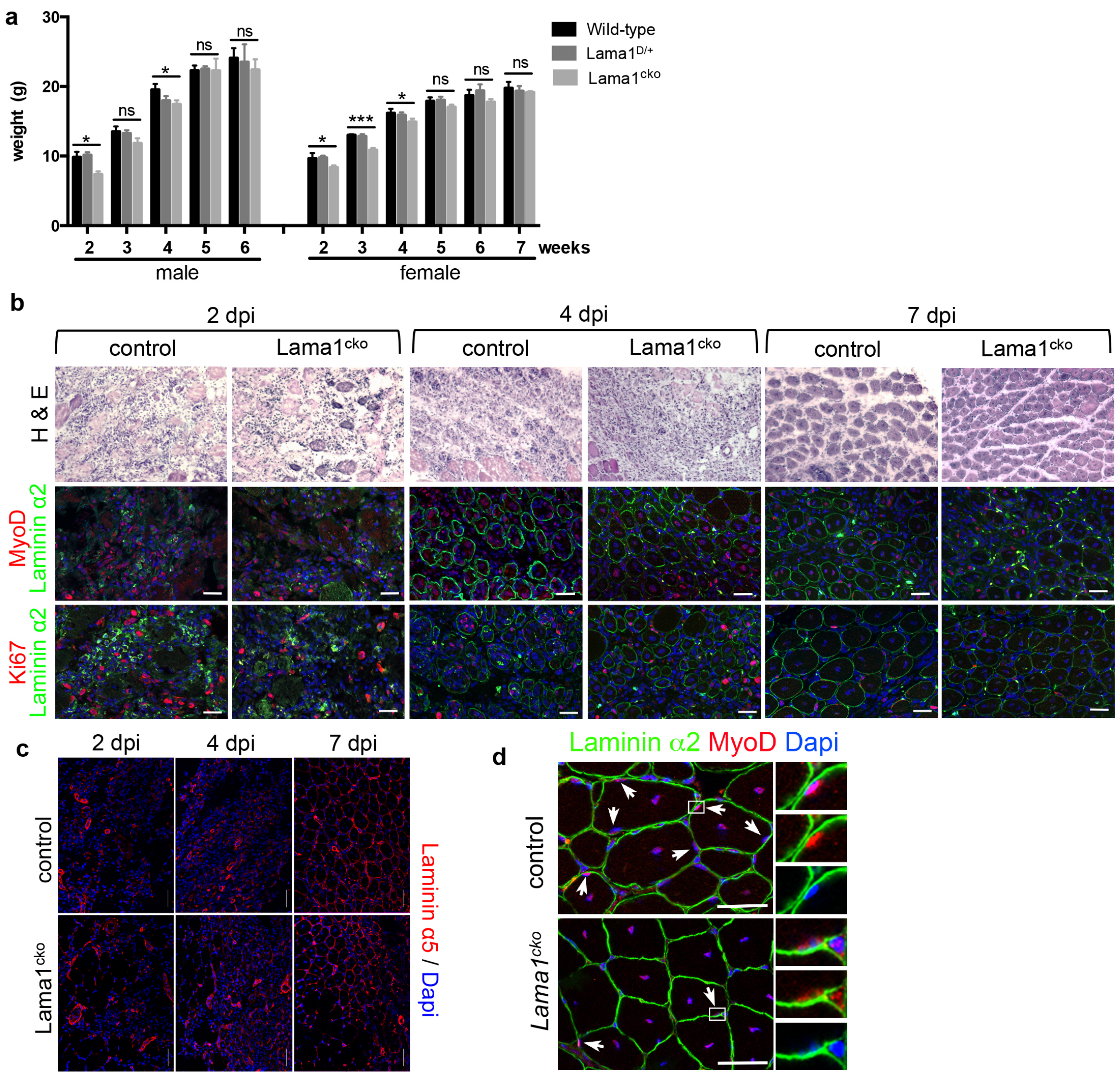

Supplementary Figure 3: Muscle regeneration is not overtly affected in *Lama1^{cko}* mice. (a) Post-natal body weight of control, heterozygotes (*Lama1$^{\Delta/+}$*) and *Lama1^{cko}* mice between 2 and 7 weeks. Error bars indicate the standard error of the mean (sem). n= 2-11 animals per time point. *P < 0.05, **P < 0.01, ***P < 0.001 (t test). (b) Muscle regeneration following cardiotoxin-mediated injury was analysed at 2, 4 and 7 days post injury using haematoxylin and eosin staining (top panels), MyoD and Laminin α2 immunofluorescence (middle panels), and Ki67 and Laminin α2 immunofluorescence (bottom panels). Representative images are shown. 2dpi: control: n= 3; *Lama1^{cko}*: n=3, 4dpi: control: n= 3; *Lama1^{cko}*: n=3, 7dpi: control: n= 3; *Lama1^{cko}*: n=3. Note the presence of smaller fibres at

7dpi in *Lama1*^{cko} muscles compared to control muscles. Scale bar: 50μm. (c) Representative images of control and *Lama1*^{cko} *Tibialis anterior* muscle transverse sections analysed at 2, 4 and 7dpi by immunofluorescence with antibodies against Laminin α5 (red). Scale bar: 50μm. (d) Representative images of SCs in sublaminal position (white arrowheads) in 14dpi control and *Lama1*^{cko} muscles. White arrowheads indicate SCs in sublaminal position. Higher magnification images are shown on the right panels. Scale bar: 50μm.

Table 1. Primers and PCR protocols for mouse genotyping

Mouse strain	Primers	Protocol
mdx	DL1577 mdx-for<br>5' GCGCGAAACTCATCAAATATGCGTGTTAGTGT 3'<br>DL1509 mdx-wtrev<br>5' GATACGCTGCTTTAATGCCTTTAGTCACTCAGATAGT TGAAGCCATTTTG 3'<br>DL1573 mdx-mutrev<br>5' CGGCCTGTCACTCAGATAGTTGAAGCCATTTTA 3'	1 cycle 95°C 2min<br>5 cycles 95°C 30sec<br>60°C 30sec<br>72°C 30sec<br>23 cycles 95°C 30sec<br>64°C 30sec<br>72°C 30sec<br>1 cycle 72°C 2min
Tg (Pax7-GFP)	SV40pA F3 – for<br>5' CCACACCTCCCCCTGAACCTGAAACATAAA 3'<br>Pax7 R10 –rev<br>5' GAATTCCCCGGGGAGTCGCATCCTGCGG 3'	1 cycle 95°C 5min<br>34 cycles 95°C 30sec<br>60°C 30sec<br>72°C 1min<br>1 cycle 72°C 10min
Tg (Sox2-Cre)	Cre-for<br>5' GCGGTCTGGCAGTAAAAACTATC 3'<br>Cre-rev<br>5' CACCAGAGACGGAAATCCATCGC 3'	1 cycle 95°C 5min<br>34 cycles 95°C 30sec<br>60°C 30sec<br>72°C 1min<br>1 cycle 72°C 10min
Lama1	Ln137<br>5' CTCGAGGTCGACGGTATCGATAAGCTTCGA 3'<br>Ln150<br>5' CCTGTTTAAAGGGCCAAACGGTACAGG 3'<br>Ln151<br>5' TCATTTTGGAAAAACTCGTTTTAAACC 3'	1 cycle 95°C 10min<br>34 cycles 95°C 30sec<br>60°C 30sec<br>72°C 1min<br>1 cycle 72°C 5min

Table 2. Primers used for qPCR

Method	Gene name	Primers sequence or reference
iCycler	*Lama1*	5'-ACCGCAGGACACTCCTGTCAGG-3'
		5'-TTACGCGCCGTCTGGTTC-3'
	Lama5	5'-ACCCAAGGACCCACCTGTAG-3'
		5'- TCATGTGTGCGTAGCCTCTC-3'
	GAPDH	5'-ACTCCACTCACGGCAAATTC-3'
		5'-GACTCCACGACATACTCAGCAC-3'
StepOne	*Lama1*	Mm01226102_m1 (Thermo Fisher Scientific)
	Lama2	Mm00550083_m1 (Thermo Fisher Scientific)
	Lama3	Mm01254735_m1 (Thermo Fisher Scientific)
	Lama4	Mm01193660_m1 (Thermo Fisher Scientific)
	Lama5	Mm01222019_m1 (Thermo Fisher Scientific)
	Lamb1	Mm00801853_m1 (Thermo Fisher Scientific)
	Lamb2	Mm00493080_m1 (Thermo Fisher Scientific)
	Lamb3	Mm00493108_m1 (Thermo Fisher Scientific)
	Lamc1	Mm00711820_m1 (Thermo Fisher Scientific)
	Lamc2	Mm00500494_m1 (Thermo Fisher Scientific)
	Lamc3	Mm01324510_m1 (Thermo Fisher Scientific)
	Pax7	Mm01354484_m1 (Thermo Fisher Scientific)
	MyoD	Mm00440387_m1 (Thermo Fisher Scientific)
